# Supplementary material for: Tumorigenicity decrease in Bcl-xL deficient MDCK cells ensuring the safety for influenza vaccine production
Source: PLoS One. 2024 Dec 16;19(12):e0311069. doi: 10.1371/journal.pone.0311069 (PMC11649150; doi:10.1371/journal.pone.0311069)
Supplement: S1 File — (ZIP) [file pone.0311069.s002.zip › S2_File/4_KOG_enrichment/scatter/B_vs_A.sign_KOG_enrichment_scatter.pdf]

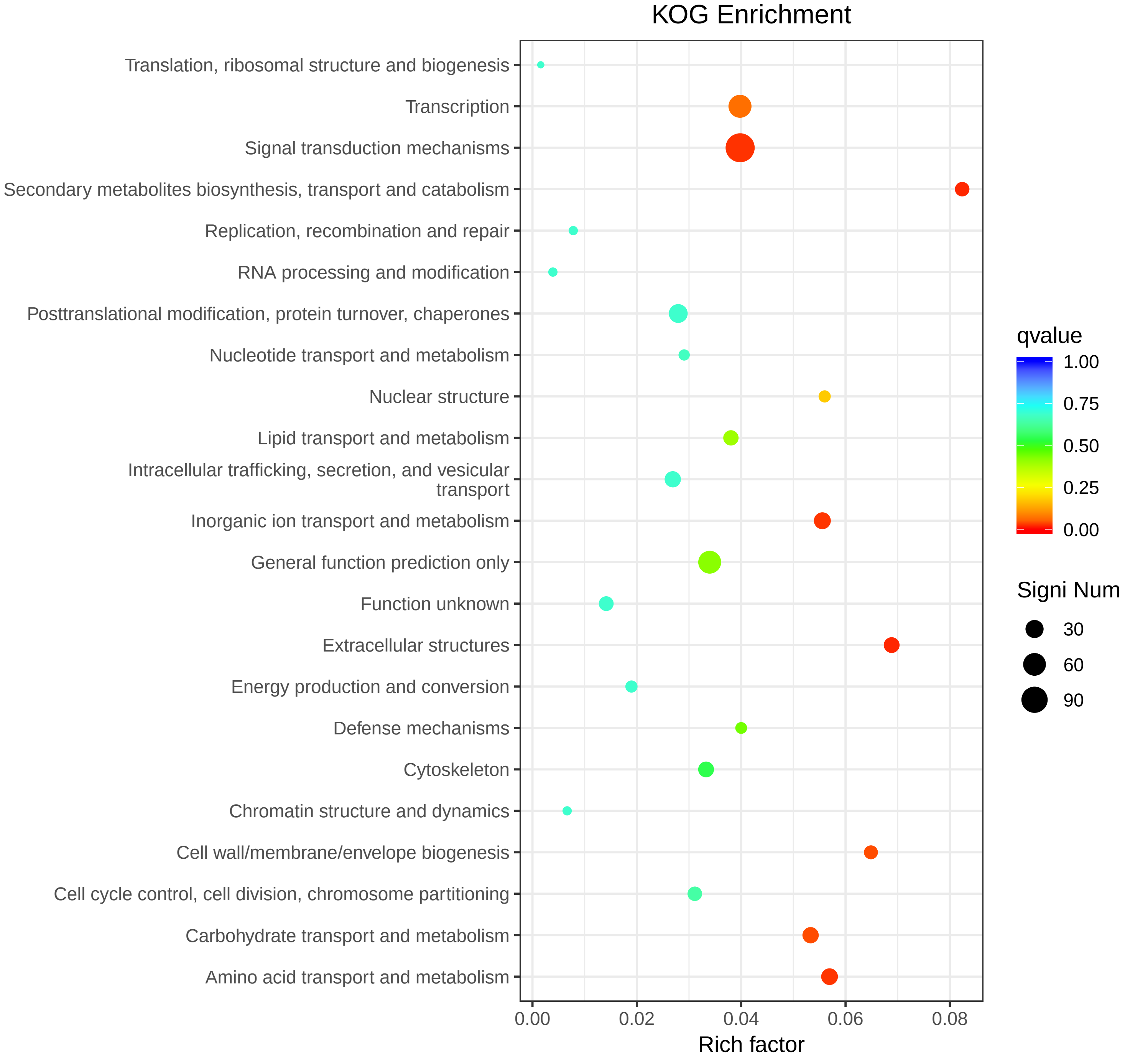
KOG Enrichment
Translation, ribosomal structure and biogenesis
Transcription
Signal transduction mechanisms
Secondary metabolites biosynthesis, transport and catabolism
Replication, recombination and repair
RNA processing and modification
Posttranslational modification, protein turnover, chaperones
Nucleotide transport and metabolism
Nuclear structure
Lipid transport and metabolism
Intracellular trafficking, secretion, and vesicular transport
Inorganic ion transport and metabolism
General function prediction only
Function unknown
Extracellular structures
Energy production and conversion
Defense mechanisms
Cytoskeleton
Chromatin structure and dynamics
Cell wall/membrane/envelope biogenesis
Cell cycle control, cell division, chromosome partitioning
Carbohydrate transport and metabolism
Amino acid transport and metabolism
0.00
0.02
0.04
0.06
0.08
Rich factor
qvalue
1.00
0.75
0.50
0.25
0.00
Signi Num
30
60
90
